# Renal denervation attenuates aldosterone expression and associated cardiovascular pathophysiology in angiotensin II-induced hypertension

**DOI:** 10.18632/oncotarget.12182

**Published:** 2016-09-21

**Authors:** Mo-Na Hong, Xiao-Dong Li, Dong-Rui Chen, Cheng-Chao Ruan, Jian-Zhong Xu, Jing Chen, Yong-Jie Wu, Yu Ma, Ding-Liang Zhu, Ping-Jin Gao

**Affiliations:** ^1^ Department of Hypertension, State Key Laboratory of Medical Genomics, Shanghai Key Laboratory of Hypertension, Ruijin Hospital, Shanghai Jiao Tong University School of Medicine, Shanghai, China; ^2^ Shanghai Institute of Hypertension, Shanghai, China; ^3^ Laboratory of Vascular Biology and Key Laboratory of Stem Cell Biology, Institute of Health Sciences, Shanghai Institutes for Biological Sciences, Chinese Academy of Sciences and Shanghai Jiao Tong University School of Medicine, Shanghai, China

**Keywords:** aldosterone, angiotensin II, remodeling, renal denervation, sympathetic nervous system, Pathology Section

## Abstract

The sympathetic nervous system interacts with the renin-angiotensin-aldosterone system (RAAS) contributing to cardiovascular diseases. In this study, we sought to determine if renal denervation (RDN) inhibits aldosterone expression and associated cardiovascular pathophysiological changes in angiotensin II (Ang II)-induced hypertension. Bilateral RDN or SHAM operation was performed before chronic 14-day Ang II subcutaneous infusion (200ng/kg/min) in male Sprague-Dawley rats. Bilateral RDN blunted Ang II-induced hypertension and ameliorated the mesenteric vascular dysfunction. Cardiovascular hypertrophy in response to Ang II was significantly attenuated by RDN as shown by histopathology and transthoracic echocardiography. Moreover, Ang II-induced vascular and myocardial inflammation and fibrosis were suppressed by RDN with concurrent decrease in fibronectin and collagen deposition, macrophage infiltration, and MCP-1 expression. Interestingly, RDN also inhibited Ang II-induced aldosterone expression in the plasma, kidney and heart. This was associated with the reduction of calcitonin gene-related peptide (CGRP) in the adrenal gland. Ang II promoted aldosterone secretion which was partly attenuated by CGRP in the adrenocortical cell line, suggesting a protective role of CGRP in this model. Activation of transforming growth factor-β (TGF-β)/Smad and mitogen-activated protein kinases (MAPKs) signaling pathway was both inhibited by RDN especially in the heart. These results suggest that the regulation of the renal sympathetic nerve in Ang II-induced hypertension and associated cardiovascular pathophysiological changes is likely mediated by aldosterone, with CGRP involvement.

## INTRODUCTION

Activation of both the renin-angiotensin-aldosterone system (RAAS) and the sympathetic nervous system (SNS) is closely associated with hypertension. Angiotensin II (Ang II) acts via the central nervous system pathway to increase sympathetic nervous activity (SNA) in the progression of hypertension [1]. Similarly, sympathetic overactivity participates in blood pressure elevation through stimulation of RAAS [2]. Therefore, there is increasing recognition that the SNS and the RAAS work in conjunction to amplify each other's action leading to hypertension [3]. However, different doses of Ang II with or without dietary salt intake produce distinct profiles of hypertension and associated changes in the sympathetic drive [4]. Whether the renal sympathetic nerve regulates Ang II-induced cardiovascular inflammation and remodeling is not fully understood.

Renal denervation (RDN) by ablation of renal sympathetic nerves was utilized as a potential treatment for patients with resistant hypertension [5-7]. Although the randomized SIMPLICITY 3 trial showed no significant difference in lowering of blood pressure between sham-treated and denervation-treated patients [8], recent clinical studies have suggested that RDN may have additional beneficial effects on diseases, such as heart failure, [9] atrial fibrillation [10] and chronic kidney disease [11]. These diseases are associated with the dysregulation of renin-angiotensin-aldosterone system (RAAS). Afferent nerve-derived calcitonin gene-related peptide (CGRP) is identified as a potent vasodilator and a hypotensive peptide [12], which has protective properties in cardiovascular disease [13, 14]. CGRP release is stimulated by mechanisms that include angiotensin II and sympathetic nerve reflexes [12, 15]. However, whether the effect of RDN on CGRP expression mediates aldosterone production is little known.

There is a strong correlation between the sympathetic nervous system and the RAAS in promoting cardiovascular disease [16]. In the present study, we aimed to test the hypothesis that bilateral RDN attenuates Ang II-induced cardiovascular dysfunction, inflammation and fibrosis, likely mediated by aldosterone possibly with CGRP involvement.

## RESULTS

### Evaluation of RDN procedure

After 2 weeks of Ang II infusion, we confirmed that RDN eliminated tyrosine hydroxylase (TH)-positive sympathetic nerve fibers in the adventitia of renal arteries by using immunofluorescence staining (Figure S2A). Western blot also showed that RDN markedly decreased TH expression in whole rat kidneys (Figure S2B). The efficacy of RDN was further confirmed by measuring NE content in kidney. Kidney NE levels were significantly reduced after RDN compared with renal innervated rats (Figure S2C).

### Effect of RDN on blood pressure and peripheral vascular relaxation and contraction

**Figure 1:**
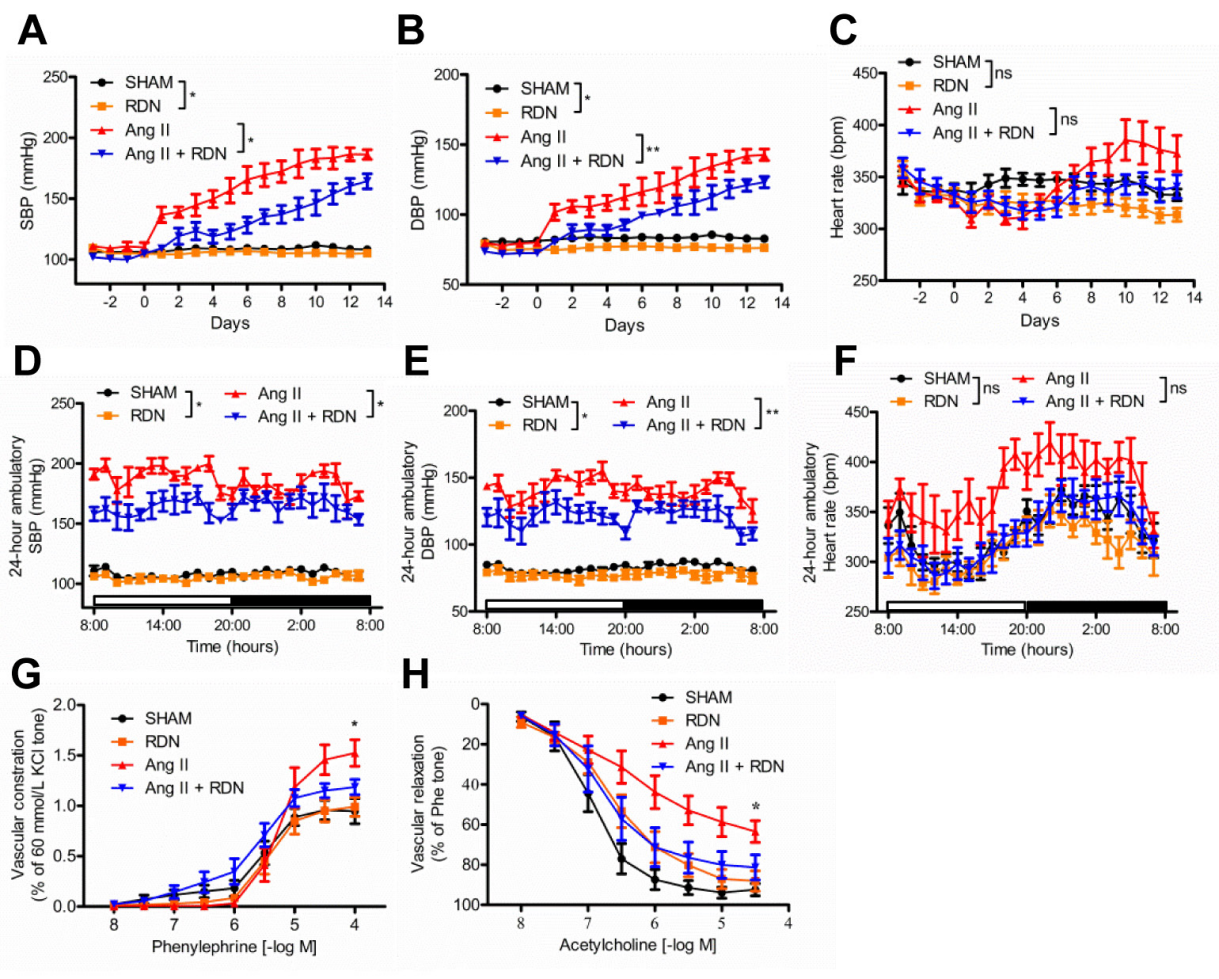
Effect of RDN on blood pressure and peripheral vascular function **A.**-**C.**, a 24-hour averaged SBP, DBP and HR over 17 consecutive days after renal denervation. (*P < 0.05, **P < 0.01, n = 5-6 in each group). **D.**-**F.**, 24-hour averaged SBP, DBP and HR during dark and light period on day 14 of Ang II infusion (*P < 0.05, **P < 0.01, n = 5-6 in each group). **G.**-**H.**, Phenylephrine-induced contraction and acetylcholine-induced endothelium-dependent relaxation in rat mesenteric arteries. n = 7 in each group. *P < 0.05.

To investigate the effect of RDN on arterial pressure, blood pressure was measured invasively by radiotelemetry. Both sham-operated and RDN-treated rats exhibited an increase in SBP and DBP during the 14-day of Ang II treatment. SBP and DBP in sham-operated rats rose at a greater rate during this period. Ang II infusion markedly increased SBP to 186.3 ± 3.925 mmHg on day 14, while in rats that previously underwent bilateral RDN, this increase was less (164.3 ± 6.176 mmHg Figure 1A and 1D, P = 0.0168). SBP was also reduced in RDN group compared with sham group (Figure 1A and 1D, 108.3 ± 1.1752 vs 104.4 ± 2.169, P < 0.05). Similarly, the DBP was also significantly reduced in RDN-treated rats (Figure 1B and 1E). Compared to Ang II group, we found a slight reduction in HR in Ang II + RDN group, which was validated by 24-hour ambulatory BP monitoring, although there was no significant difference (Figure 1C and 1F). To further determine the vascular function, mesenteric arteries were isolated for vascular ring assay. Ang II-induced hypertension was associated with blunted acetylcholine-induced endothelium-dependent relaxation which was ameliorated by RDN (Figure 1G). Similarly, Ang II markedly augmented contraction to phenylephrine which was attenuated by RDN (Figure 1H).

### Effect of RDN on cardiac remodeling and fibrosis

**Figure 2:**
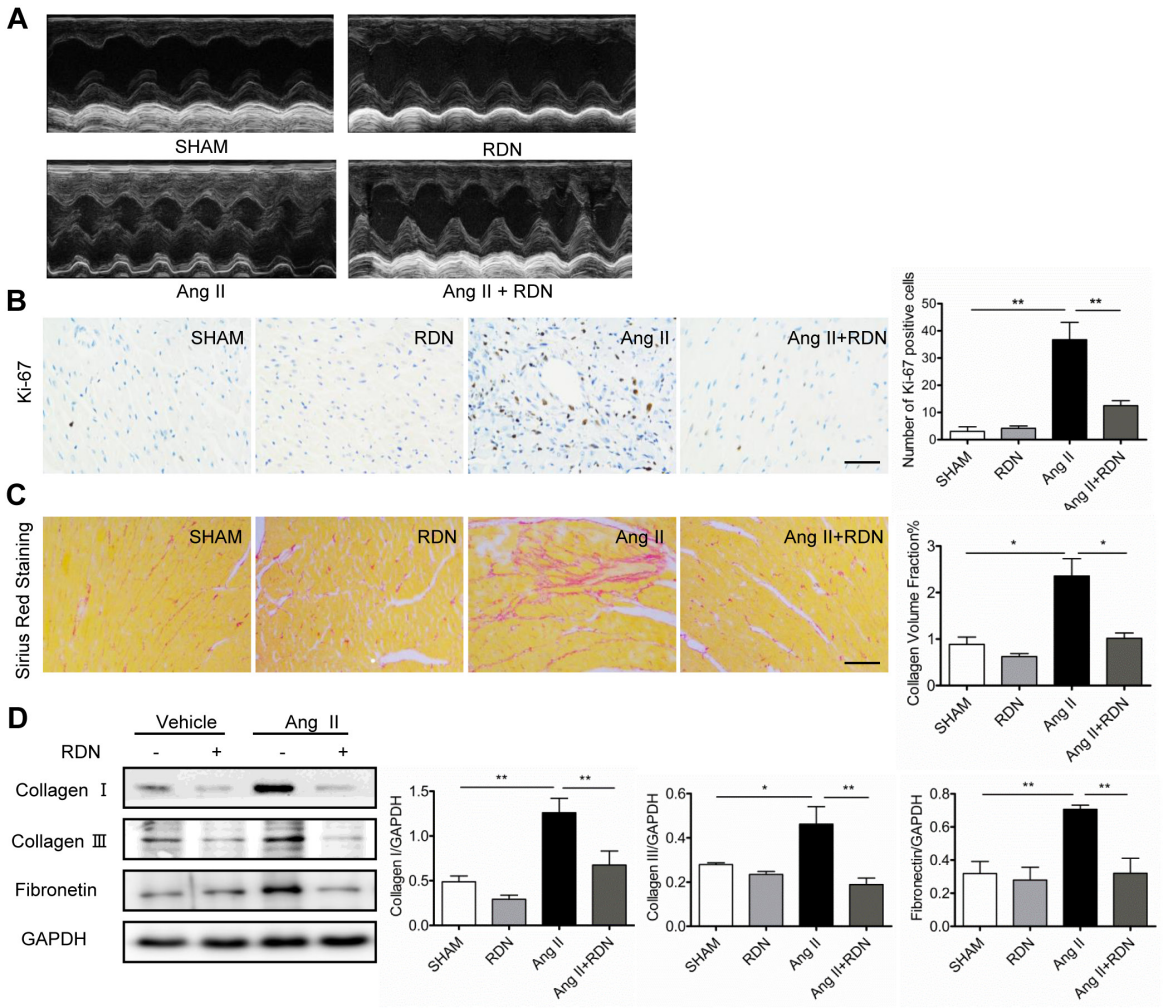
RDN prevents cardiac remodeling and fibrosis **A.** Echocardiograms of the left ventricle in long-axis M mode view, left ventricular (LV) internal dimension and intraventricular septum in both systole and diastole. Various parameters of cardiac function were also measured (Table 1). **B.** Ki-67 immunostaining in heart sections. Scale bar represents 100 μm. **C.** Heart sections were stained and quantified for collagen accumulation. Scale bar represents 200 μm. **D.** Cardiac protein expression of Collagen I, Collagen III, Fibronectin. Anti-GAPDH antibody served as a loading control. Values are mean ± SEM. n = 3-8 in each group. *P < 0.05, **P < 0.01.

Ang II-induced cardiac hypertrophy was significantly blunted in rats which had previously undergone RDN. Cardiac structural and functional changes were measured by echocardiography 13 days after the onset of Ang II infusion (Figure 2A, Table 1). Ang II significantly increased LV wall thickness, as estimated by echocardiography and heart weight (HW)/body weight (BW) ratio, and these hypertrophic responses to Ang II stimulation were ameliorated by RDN. No changes of LVEF or LVFS were seen in each group. In Figure 2B, ki-67-positive cells were dramatically increased in heart sections of Ang II-infused rats, while RDN effectively decreased the number of ki-67-positive cells. In addition, Ang II infusion caused collagen deposition in the myocardial interstitial, as evidenced by Sirius red staining. RDN significantly suppressed collagen accumulation (Figure 2C) and the expressions of collagen I/III and fibronectin (Figure 2D).

**Table 1:** Echocardiographic Analysis of Cardiac Hypertrophy and Function

	SHAM *n* = 6	RDN *n* = 6	Ang II *n* = 6	Ang II + RDN *n* = 7
hw/bw (mg/g)	2.871±0.100	2.919±0.082	3.650±0.093**	3.076±0.096† †
IVS;d (mm)	1.707±0.090	1.476±0.058	2.202±0.120**	1.843±0.091†
IVS;s (mm)	3.320±0.146	2.733±0.119	3.920±0.128	3.465±0.224
LVID;d (mm)	6.755±0.278	6.996±0.460	5.803±0.207	6.404±0.244
LVID;s (mm)	3.307±0.259	3.807±0.390	2.311±0.292	2.708±0.176
LVPW;d (mm)	1.804±0.109	1.50 ±0.071	2.268±0.113*	1.915±0.099†
LVPW;s (mm)	3.387±0.164	2.773±0.089	4.023±0.138*	3.503±0.215†
LVEF (%)	81.17±1.997	75.55±3.119	88.00±3.147	85.53±2.797
LVFS (%)	51.33±2.079	46.18±3.344	60.54±3.908	57.07±3.681

hw indicates heart weight; d, diastole; s, systole; IVS, intraventricular septum (mm); LVID, left ventricular internal dimension (mm); LVPW, LV posterior wall (mm); LVEF, left ventricular ejection fraction; LVFS, left ventricular fractional shortening. The parameters listed in the table were measured using M-mode images in long-axis views as described in the methods. Results are presented as mean ± SEM.

* *P* < 0.05

** *P* < 0.01 Ang II vs SHAM.

† *P* < 0.05

†† *P* < 0.01 Ang II + RDN vs Ang II. *n* = 6-7 for each group.

### Effect of RDN on vascular remodeling

**Figure 3:**
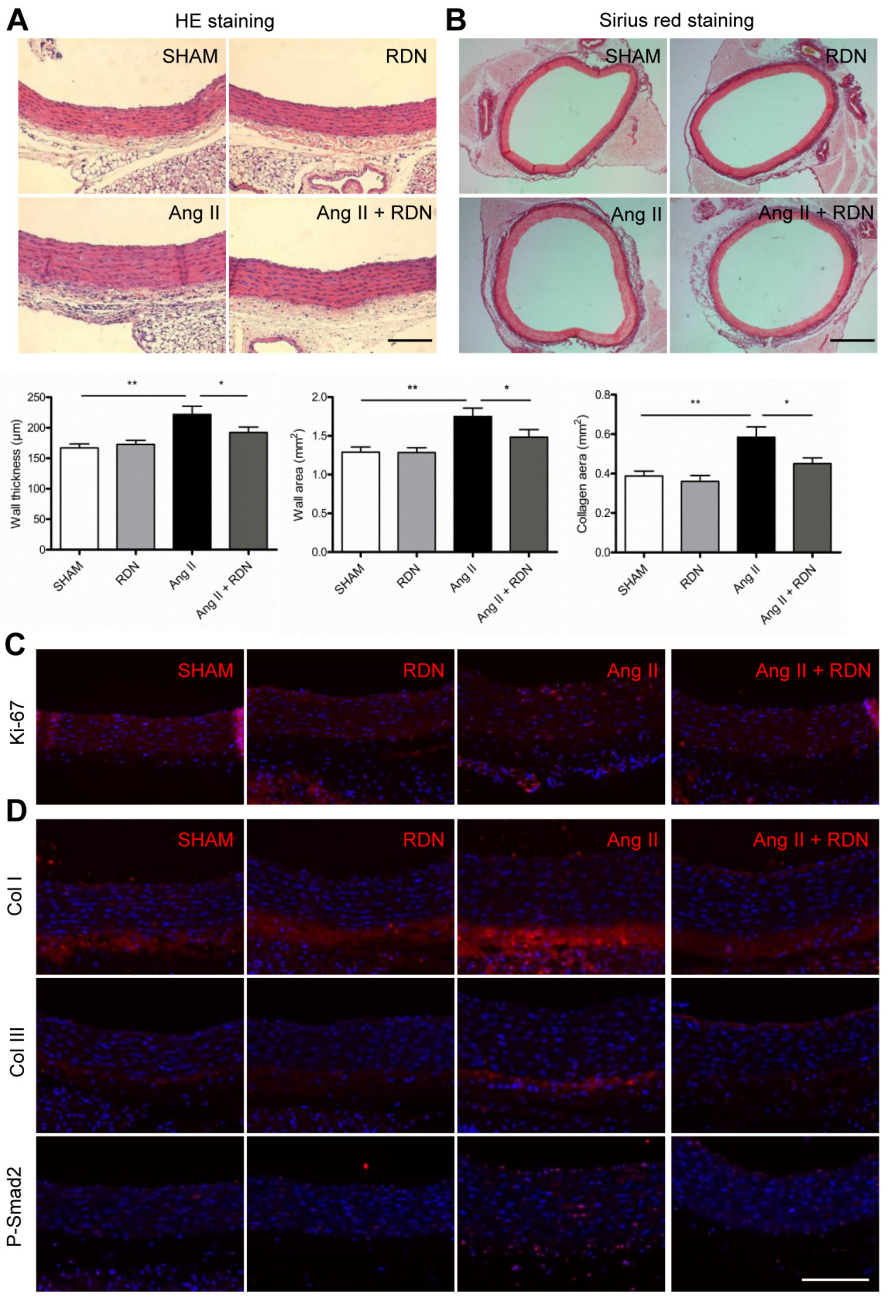
RDN suppresses vascular remodeling **A.** Quantitative histomorphometry results of vascular wall thickness and wall area. Scale bar represents 200 μm. **B.** Aorta sections were stained and quantified for collagen deposition. Scale bar represents 800 μm. **C.** Ki-67 immunostaining in aorta sections. Scale bar represents 200 μm. **D.** Immunofluorescence staining for Collagen I, Collagen III, P-Smad2 in aortas. Scale bar represents 200 μm. Values are mean ± SEM. *n* = 6-8 in each group. *P < 0.05, **P < 0.01.

To evaluate the role of renal sympathetic nerves in vascular remodeling, aortas were isolated for histomorphometry analysis. The quantitative results of vascular wall thickness and wall area of thoracic aortas are shown in Figure 3A. RDN significantly attenuated aortic wall thickness induced by Ang II. Sirius red staining identified that RDN also inhibited Ang II-accelerated perivascular fibrosis of aortas (Figure 3B). We then investigated the effect of RDN on the proliferation of vascular wall after Ang II infusion. The number of Ki-67-positive cells dramatically increased in aortas which were reduced by RDN (Figure 3C). Immunofluorescent staining also confirmed that RDN suppressed collagen I/III expression and the increase of p-Smad2-positive cells (Figure 3D).

### Effect of RDN on cardiovascular inflammation

**Figure 4:**
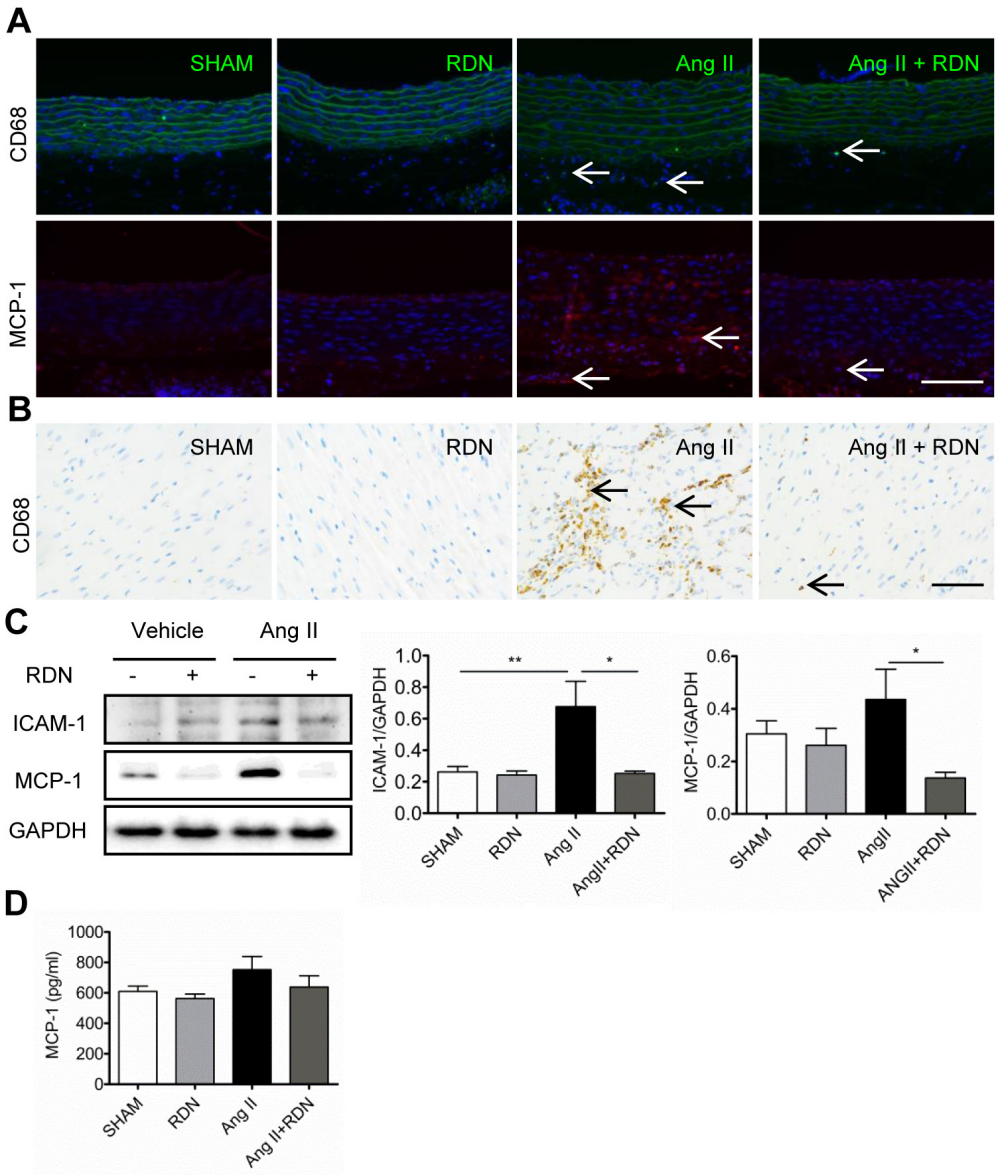
RDN inhibits cardiovascular inflammation **A.**-**B.** Representative photomicrographs of macrophage infiltration and MCP-1 expression in aorta. Scale bar represents 200 μm. **C.** Representative immunohistology images of CD68 positive cells in heart sections. Scale bar represents 50 μm. **D.** Western blot and quantification of cardiac ICAM-1 and MCP-1 protein expression. Anti-GAPDH antibody served as a loading control. **E.** Plasma levels of MCP-1 in each group measured by ELISA. n = 3-9 in each group. *P < 0.05, **P < 0.01.

Next, we examined macrophage infiltration into injured arteries and hearts by detecting CD68-positive cells. As apparent in Figure 4A and 4C, macrophages were predominantly localized in the adventitia of aortas and myocardial interstitial in Ang II group. However, RDN dramatically reduced macrophage accumulation, indicating that renal innervation may be involved in macrophage infiltration. We also found that RDN effectively suppressed MCP-1 expression in aortas (Figure 4B). In hearts, inflammatory factors, including MCP-1 and intercellular adhesion molecule 1 (ICAM-1) were also markedly reduced in Ang II + RDN group compared with Ang II group (Figure 4D). The circulating plasma level of MCP-1 was slightly decreased in Ang II + RDN group, but without statistical significance (Figure 4E).

### Effect of RDN on the level of aldosterone and underlying regulatory mechanism

**Figure 5:**
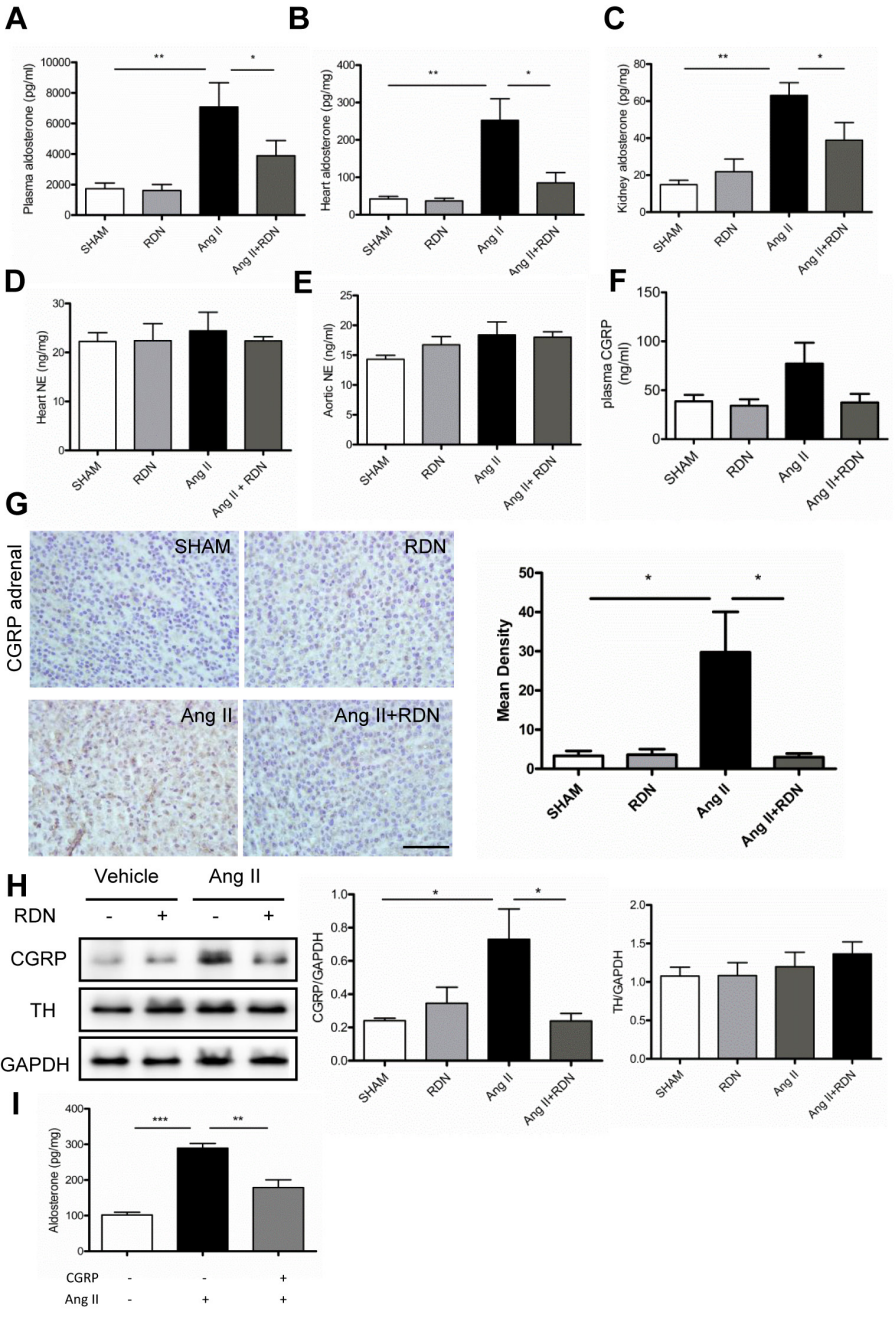
Effect of RDN on the level of Aldosterone and underlying regulatory mechanism **A.**-**C.**, plasma, cardiac and renal levels of aldosterone measured by radioimmunoassay. **D.**-**E.**, Heart and aortic NE levels in each group. **F.** The plasma CGRP level in each group. **G.** CGRP immunochemistry staining in adrenal medulla. Scale bar represents 50 μm. Values are mean ± SEM. *n* = 4-7 in each group. *P < 0.05. **H.** Western blot and quantification of CGRP and TH in the adrenal. I, CGRP (10-7 mol/L) inhibited Ang II (10-8 mol/L) induced Aldosterone secretion which wasmeasured in the H295R cell supernatant. Values are mean ± SEM, *P < 0.05, **P < 0.01,*** < 0.001.

To investigate whether RDN has impact on the components of RAAS, plasma and tissue levels of renin and aldosterone were evaluated. Interestingly, RDN significantly reduced the Ang II-induced elevation of aldosterone expression in the plasma, heart and kidney (Figure 5A-5C). However, plasma renin levels were strikingly decreased in rats with Ang II stimulation, with no significant difference between Ang II and Ang II + RDN group (Figure S3A). Plasma renin activity and tissue levels of renin were similar among these groups (Figure S3B-D). No differences of NE levels were observed in the heart and aorta (Figure 5D-5E). However, our data suggested that RDN lowered the plasma CGRP which was increased by Ang II treatment, although the differences were not statistically significant (Figure 5F). Immunochemistry staining further demonstrated that RDN inhibited the expression of CGRP in adrenal glands which was increased after Ang II infusion (Figure 5G-5H). *In vitro* study using H295R cell line, we found that CGRP lowered the Ang II-stimulated aldosterone secretion, suggesting that CGRP plays a protective role in regulating the synthesis of aldosterone (Figure 5I).

### Effect of RDN on TGF-β/Smad and mitogen-activated protein kinases (MAPKs) activation

**Figure 6:**
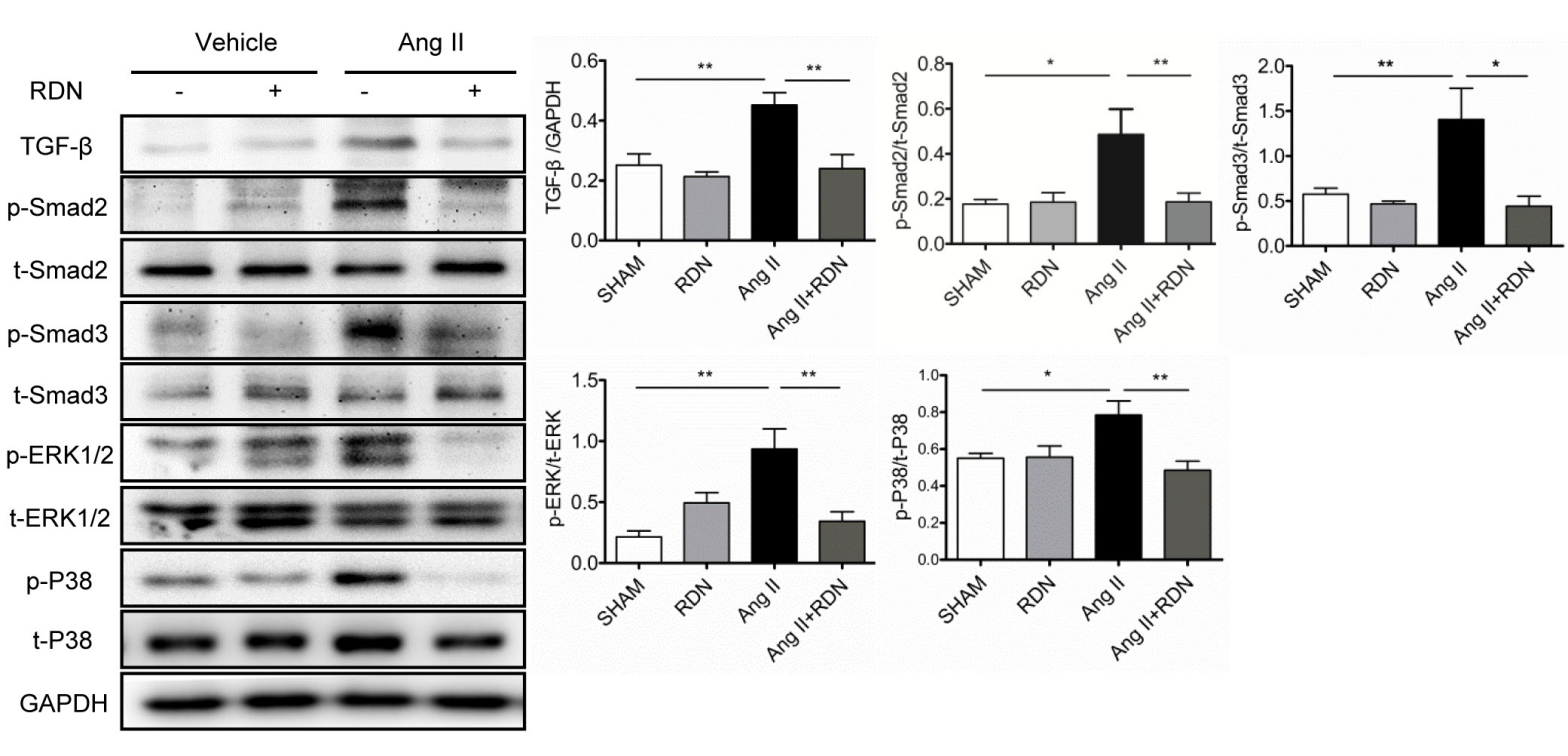
Suppression of TGF-β-Smad2/3 and MAPKs in RDN rats with Ang II stimulation Cardiac protein expression of TGF-β, Smad2 phosphorylation, Smad3 phosphorylation, ERK1/2 phosphorylation and p38 phosphorylation. Anti-GAPDH antibody served as a loading control. Values are mean ± SEM. *n* = 4-8 in each group. *P < 0.05, **P < 0.01.

To clarify the mechanism of RDN in the prevention of AngII-induced cardiac remodeling, we examined the effects of RDN on cardiac TGF-β/Smad signaling and mitogen-activated protein kinases (MAPKs) activation in Ang II-infused rats (Figure 6). Ang II stimulation caused a marked enhancement in cardiac TGF-β expression and phosphorylation of Smad2 and Smad3. However, RDN significantly attenuated these changes caused by Ang II. Likewise, RDN also inhibited extracellular signal-regulated kinases (ERK) 1/2 and p38MAPK activation induced by Ang II.

## DISCUSSION

**Figure 7:**
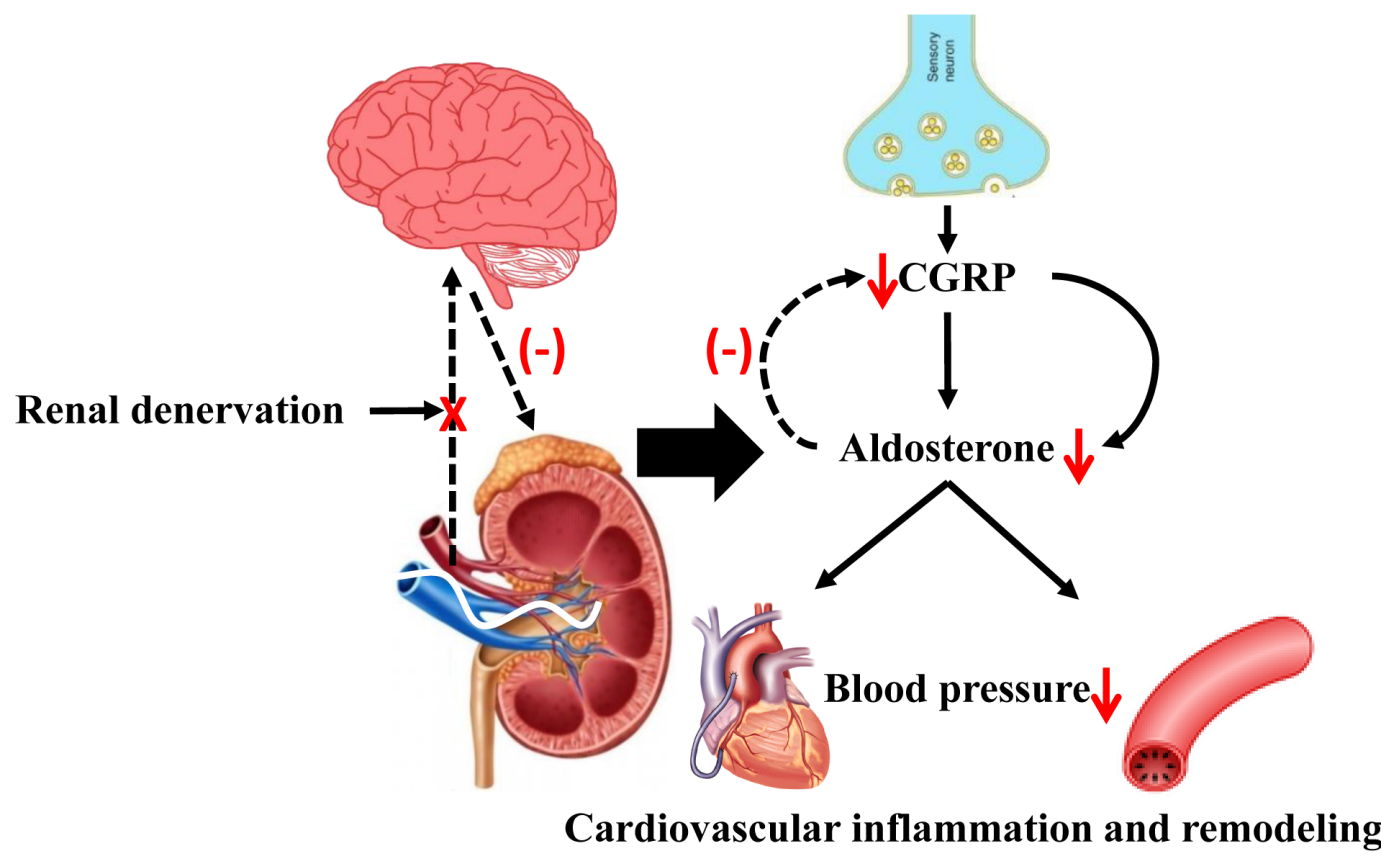
The possible role of RDN in attenuating Ang II-induced cardiovascular remodeling through inhibition of aldosterone expression Ang II promoted aldosterone expression contributing to hypertension and associated cardiovascular pathophysiology. RDN effectively inhibited aldosterone expression likely via blockade of signal between peripheral and central sympathetic nervous system with CGRP involvement, thus attenuating the cardiovascular dysfunction. Ang II indicates angiotensin II. CGRP indicates calcitonin gene-related peptide.

In the present study, we show that Ang II-induced elevation of blood pressure and peripheral vascular resistance, and associated cardiovascular pathophysiological changes was attenuated by RDN, which was likely mediated by aldosterone with CGRP involvement (Figure 7). Therefore, our data indicated that RDN seems to be a promising therapeutic strategy for RAAS activation-related cardiovascular diseases.

It is well known that Ang II infusion in experimental animals causes an increase in aldosterone production. [17, 18] The important finding in this study is that RDN effectively suppressed Ang II-induced aldosterone expression not only in the kidney and heart but also in plasma. However, RDN showed no effect on local renin and plasma renin activity which was inhibited by Ang II due to the negative feedback effect of Ang II [19]. These results suggest that aldosterone regulation is related with the renal sympathetic nerve and may be independent of renin in this model. There is an interaction between aldosterone and the autonomic nervous system that promote cardiovascular diseases. RDN employed in the present work ablates both afferent and efferent renal sympathetic nerves which regulate the peripheral sympathetic reflex. It is reported that the renal afferent nerve, but not efferent nerve, may regulate the peripheral sympathetic reflex. And cardiac afferent sympathetic deletion ameliorates cardiovascular remodeling and dysfunction in heart failure. In keeping with this, Interestingly, we further found that RDN suppressed Ang II-induced CGRP expression, but have no effect on TH expression in the adrenal gland. CGRP does not play a primary role in the regulation of basal blood pressure in normal individuals, but is suggested to have protective properties in cardiovascular disease [12, 20]. Therefore, Ang II-induced increase in CGRP might be a response to the Ang II vasoconstrictor effect [21] and contribute to the inhibition of aldosterone synthesis in the adrenocortical cell line. Masuda et al. found that CGRP levels were significantly higher than in normal controls in hypertensive patients, especially those with primary aldosteronism. However, a significant decrease in CGRP levels and a marked reduction in blood pressure were observed after adrenalectomy suggesting that CGRP levels in hypertensive patients could be a compensatory reaction to the change of blood pressure [22]. Ang II also stimulates aldosterone production through Ang II type I receptor (AT1) which was decreased by RDN in several cardiovascular models [23-25]. These data suggest that aldosterone expression regulated by AT1 receptor can cause compensatory reaction of CGRP. This might reasonably explain why RDN decreases CGRP expression associated with reduction of aldosterone expression. These results suggest that the renal sympathetic nerve regulates aldosterone secretion possibly through the protective effect of CGRP. Furthermore, we observed that RDN suppressed the increase of circulating plasma CGRP level during 14-day Ang II infusion, although the difference is not statistically significant. This is in accordance with the findings of Smillie and colleagues [20]. However, Smillie et al further found that CGRP plasma levels were significantly increased during 28-day Ang II infusion, suggesting a long term effect of Ang II on CGRP release.

Aldosterone provoked the expression of hypertrophic markers that mediate cardiovascular hypertrophy and dysfunction [26, 27]. We found that RDN decreased Ang II-induced cardiac hypertrophy, as indicated by the decrease of both systolic and diastolic LVPM as well as diastolic intraventricular septum thickness. Ablation of renal sympathetic nerves also inhibited the increase of vascular wall thickness and wall area caused by Ang II. These effects are associated with the decrease of cell proliferation in the heart and aorta [28]. Ang II infusion enhanced ECM expression as indicated by the increase of collagen and fibronectin deposition which was also markedly suppressed by RDN. Therefore, these results suggest that RDN inhibited aldosterone expression which mediated the Ang II-induced excessive tissue fibrosis leading to tissue remodeling, a contributing factor to cardiovascular diseases.

It is possible that many of the beneficial effects of RDN are simply related to a decrease in blood pressure and the resultant decrease in pressure-induced cardiovascular damages [29]. An apparent hypotensive effect of RDN in Ang II-induced hypertension was observed using telemetry methods. This effect was associated with improvement of mesenteric artery relaxation and contraction. Aldosterone induces contraction and endothelial dysfunction especially in the resistance arteries [30, 31]. Therefore, the beneficial effect of RDN on vascular function may be through reduction of aldosterone expression. Although systolic blood pressure still exceeded 160mmHg, Ang II promoted cardiovascular inflammatory responses like macrophage infiltration and proinflammatory cytokines expression as well as cardiovascular remodeling was significantly normalized by RDN. By employing unilateral denervation, Xiao et al [29] found that renal inflammation was reduced in the denervated, but not in the innervated kidney in Ang II-induced hypertension. Other studies also showed that RDN has protective effects on the vasculature and heart independent of blood pressure lowering [32, 33]. As mentioned above, we found that RDN attenuated Ang II-induced systemic and local aldosterone production. Virdis's study also found that aldosterone increased SBP was partially reversed by spironolactone, a mineralocorticoid receptor antagonist. However, the media/lumen ratio and impaired response to acetylcholine were normalized by spironolactone, suggesting RDN may attenuate vascular remodeling through decrease of local aldosterone [34]. However, high blood pressure-caused shear stress and stretch also promotes inflammatory response [35, 36], suggesting that cardiovascular pathophysiological changes after RDN may be partly dependent on blood pressure in the Ang II infusion model.

In conclusion, we found that the renal sympathetic nerve regulates aldosterone expression associated cardiovascular dysfunction, inflammation and fibrosis in Ang II-infused rats.likely via CGRP suggesting that RDN had additional benefits on hypertensive target organ damages. However, further investigation is required to explore the potential of CGRP to be an effective approach to treat RAAS-related cardiovascular diseases.

## MATERIALS AND METHODS

### Experimental animals

Male Sprague-Dawley rats weighing 300-350 g were purchased from Shanghai Slac Laboratory Animal Company. All rats were housed under a 12-hour day/night cycle with free access to standard chow and water. All rat experiments were performed in accordance with guidelines for the Care and Use of animals established by Shanghai Jiao Tong University School of Medicine.

First, all rats were divided into 2 groups, including sham-operated rats and renal denervated rats. RDN was performed as previously described [37]. Briefly, rats were intraperitoneal anesthetized with sodium pentobarbital (50 mg/Kg) and a ventral midline incision was made. RDN was performed surgically by cutting all of the visible nerves of renal artery and vein after isolating the vessels from the surrounding adipose and connective tissues. Then the vessels were coated with a solution of 10% phenol in ethanol. Sham-operated rats underwent the same surgical procedure without isolating vessels or stripping of the renal nerves. Blood pressure was measured invasively using telemetry devices (TA11PA-C40; Data Science International, St Paul, MN) in the abdominal cavity [38].

After one week of recovery, sham-operated and RDN groups were further randomized into 2 groups, respectively. An osmotic minipump (Model 2002, Alzet, Palo, Alto, CA) was implanted subcutaneously with Ang II (200 ng/kg/min, CalBiochem, La Jolla, CA) or vehicle for 14 days. Therefore, the groups were as follows: vehicle-treated sham rats (sham, n = 12), vehicle-treated RDN rats (RDN, n = 12), Ang II-treated sham rats (Ang II, n = 12) and Ang II-treated RDN rats (Ang II + RDN, n = 12).

A timeline of animal procedure was presented in the Figure S1.

### Verification of RDN

Achievement of RDN was assessed by detecting renal norepinephrine (NE) content using an NE ELISA kit (LDN, Nordhorn, Germany) according to manufacturer's instructions. Briefly, kidneys were homogenized in 0.01N HCl containing EDTA (1 mmol/L) and sodium metabisulfite (4 mmol/L) followed by 10 minutes centrifugation at 18000g, the supernatant was then collected. Similarly, NE was also measured in the heart and aorta.

For immunofluorescence staining of nerve fibers, renal artery sections were incubated with primary antibodies against tyrosine hydroxylase (TH, EMD Millipore, Billerica, MA), a marker of sympathetic nerves, followed by fluorescence-conjugated secondary antibody (Life Technologies) using Carl Zeiss microscope. In addition, the expression of TH in renal extracted protein was determined by western blot.

### Echocardiography

At 13 days after Ang II infusion, transthoracic echocardiography was performed under anesthesia with isoflurane with a Vevo 2100 high-resolution imaging system equipped with a 21-MHz transducer (Visualsonics Toronto, ON, Canada). The recording values include the left ventricular internal diameter (LVID), interventricular septum thickness (IVST), left ventricular posterior wall (LVPW), left ventricular fractional shortening (LVFS) and left ventricular ejection fraction (LVEF). The analysis was conducted in a blinded manner in the long-axis view from M-mode recordings at end diastole and end systole stage.

### Vessel preparation and measurement of isometric force in rat vascular rings

As described previously [39], the third branch of rat mesenteric arteries were dissected with special care for the preservation of endothelium, cut into 1-2 mm rings and mounted on isometric force transducers (Danish Myo Technology Model 610 M, Denmark). The organ bath was filled with 5 mL Kreb's solution (pH 7.4; NaCl, 121 mmol/L; KCl, 5.9 mmol/L; CaCl_2_, 2.5 mmol/L; MgCl_2_, 1.2 mmol/L; NaH_2_PO_4_, 1.2 mmol/L; NaHCO_3_, 15.5 mmol/L and D-glucose, 11.5 mmol/L), aerated with 95% O_2_ and 5% CO_2_ under an initial resting tension of 1mN at 37 °C. Force was recorded via a PowerLab/8sp data acquisition system (A.D. Instruments, Castle Hill, Australia). Both acetylcholine-induced vasodilation and phenylephrine-induced vasoconstriction were evaluated.

### Measurement of plasma renin, renin activity, aldosterone, CGRP and MCP-1

Plasma samples were collected via heart puncture in chilled tubes containing EDTA 2 weeks after Ang II infusion. Plasma renin activity (Sigma, St. Louis, MO), renin (Molecular Innovations, Novi, MI), CGRP (Phoenix, USA) and monocyte chemoattractant protein-1 (MCP-1, Antigenix, NY, USA) were measured by ELISA according to manufacturer's instructions. Plasma aldosterone was measured using radioimmunoassay in the lab of Shanghai Institute of Endocrinology.

### Kidney and heart contents of renin and aldosterone

100 mg kidney and heart were homogenized in 1 mL PBS followed by centrifugation at 18000g for 10 minutes. Then supernatant was collected to measure the content of renin by ELISA kit and aldosterone by radioimmunoassay.

### Histology and immunohistochemistry

Paraffin-embedded rat hearts, aortas and adrenal were sectioned 5 um thick. Aorta sections were stained with hematoxylin and eosin (HE, Maixin, Fuzhou, China) for the analysis of histomorphometry. For measurement of collagen volume fraction, sections were stained with Sirius red (0.5% wt/vol in saturated aqueous picric acid; Leagene, China). The positive fibrosis area per field was evaluated by image analysis software (Image J, National Institutes of Health, Bethesda, MD, USA). Immunohistochemistry or immunofluorescence were performed as previously described [40] with primary antibodies against TH (EMD Millipore, Billerica, MA), CD68 (Abcam, Cambridge, MA), Ki-67 (Anaspec, CA, USA), Collagen I (EMD Millipore), Collagen III (Abcam, Cambridge, MA), CGRP (Biorbyt, UK), MCP-1 (Abcam, Cambridge, MA) and phosphor-Smad2 (CST, Danvers, MA, USA). Then the sections were incubated to FITC-conjugated (Life Technologies, Invitrogen, Carlsbad, CA) or horseradish peroxidase-conjugated (Santa Cruz, CA, USA) secondary antibodies.

### Western blot

Western blot analysis was performed as previously described [40]. Primary antibodies were Collagen I (EMD Millipore), Collagen III (Abcam), Fibronectin (PTG, CHI, USA), ICAM-1 (Abcam), MCP-1 (Abcam), CGRP (Biorbyt, UK) and GAPDH (PTG). ERK 1/2 (44/42kD), phospho-ERK 1/2 (44/42kD), P38-MAPK, phosphor-P38-MAPK, TGF-β, Smad2, phosphor-Smad2, Smad3, phospho-Smad3 antibodies were obtained from Cell signaling technology (CST, Danvers, MA, USA). Secondary antibodies were obtained from Santa Cruz Biotechnology. All experiments were done with at least 3 replications.

### Cell culture

Adrenocortical carcinoma H295R cells was purchased from ATCC and were cultured in DMEM/F12 (Gibco) supplemented with 2.5% nu-serum replacement (BD), 1% ITS+Premix (BD), and 1% L-glutamine (Gibco). After cells were stimulated by either Ang II and CGRP for 24 hours, the aldosterone concentration in the supernatant was measured by radioimmunoassay and normalized to total cell protein.

### Data analysis

Data were expressed as mean ± SEM. All statistical analyses were performed with Graphpad software (Version 5.01). Comparison between two groups was calculated by t-test and across multiple groups by one-way ANOVA using the Newman-Keuls method. A probability value of *P* < 0.05 was considered statistically significant.

## SUPPLEMENTARY MATERIALS FIGURES


