# Supplementary material for: Renal denervation attenuates aldosterone expression and associated cardiovascular pathophysiology in angiotensin II-induced hypertension
Source: Oncotarget. 2016 Sep 21;7(42):67828–40. doi: 10.18632/oncotarget.12182 (PMC5356522; doi:10.18632/oncotarget.12182)
Supplement: Supplementary file 1 [file oncotarget-07-67828-s001.pdf]

Renal denervation attenuates aldosterone expression and associated cardiovascular pathophysiology in angiotensin II-induced hypertension

Supplementary Material

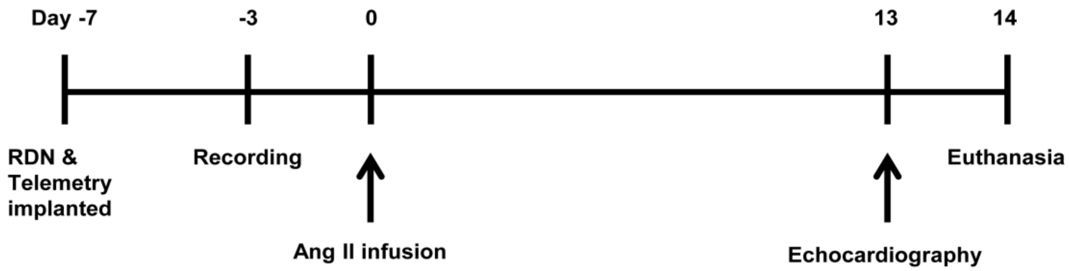

Figure S1: A timeliness of animal procedure.

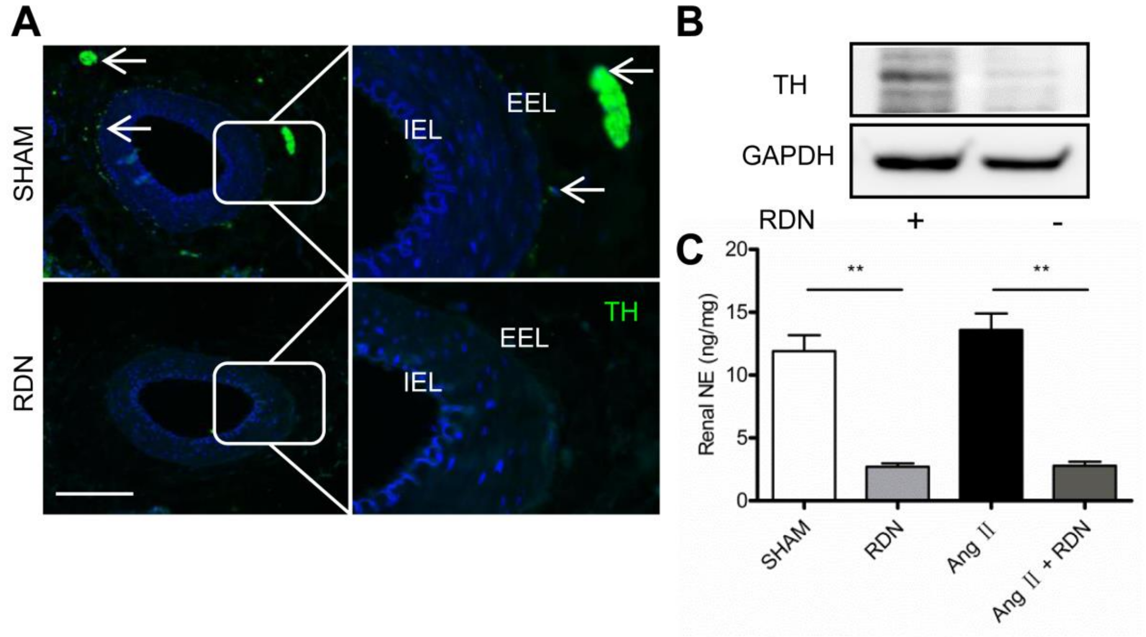

Figure S2. RDN abolishes TH expression and NE level in kidney. A, Immunofluorescence of tyrosine hydroxylase (TH) in denervated or innervated renal arteries. Scale bar represents 200 μm. B, TH expression in renal denervation (RDN) or intact kidneys using Western blot analysis. RDN doesn't affect the TH expression in adrenal. C, NE level in RDN or intact kidneys using ELISA. Data were presented as mean ± SEM. n=5-8 in each group, ***P*<0.01. IEL indicates internal elastic lamina; EEL indicates external elastic lamina.

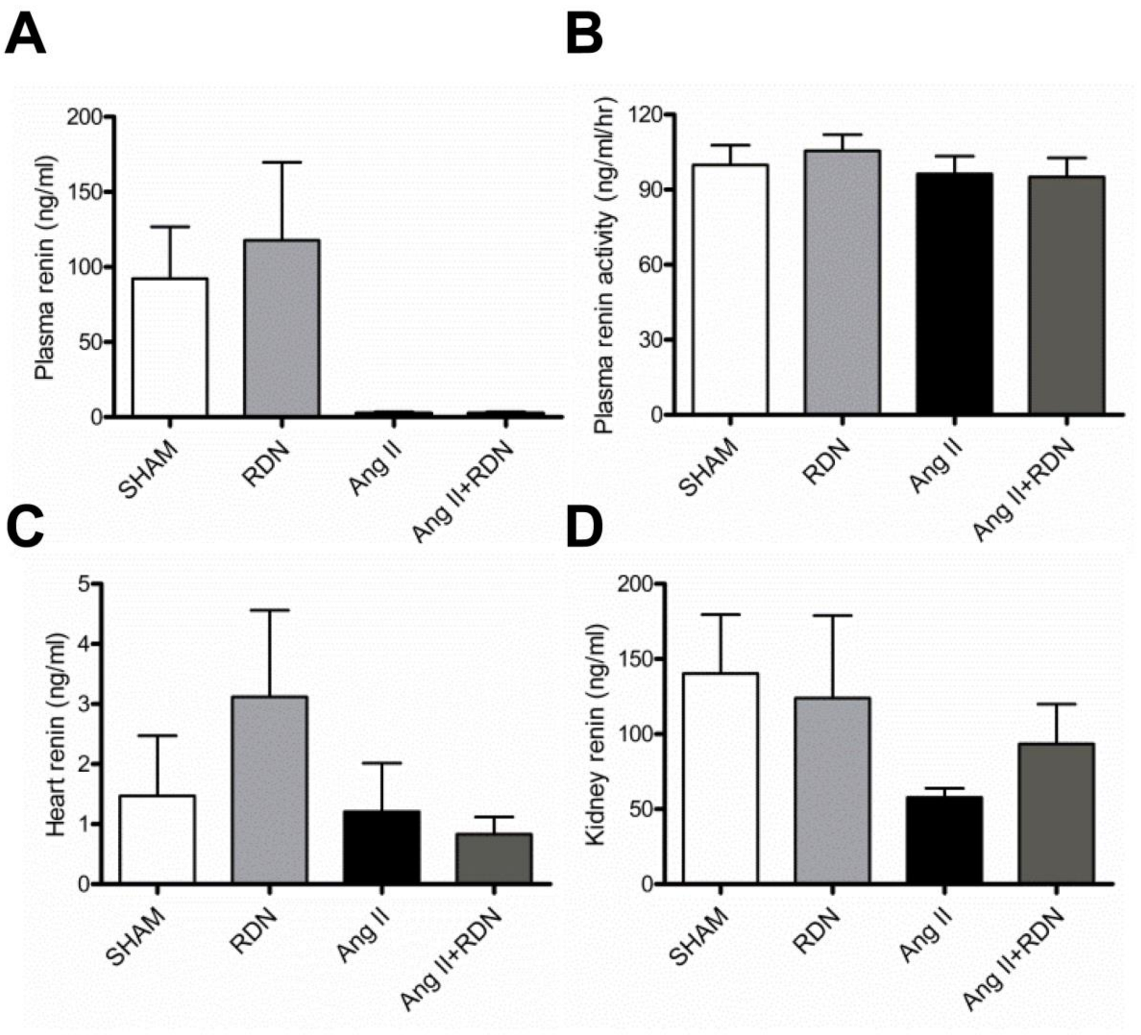

Figure S3: Effect of RDN on renin and plasma renin activity. A Plasma renin levels in sham, RDN, Ang II and Ang II+RDN. B, Plasma renin activity in sham, RDN, Ang II and Ang II+RDN. C-D, Kidney and heart levels of renin, respectively. Values are mean ± SEM. n=4-8 in each group. **P*<0.05, ***P*<0.01.
